# A Review of Groundwater Arsenic Contamination in Bangladesh: The Millennium Development Goal Era and Beyond

**DOI:** 10.3390/ijerph13020215

**Published:** 2016-02-15

**Authors:** Fakir Md. Yunus, Safayet Khan, Priyanka Chowdhury, Abul Hasnat Milton, Sumaira Hussain, Mahfuzar Rahman

**Affiliations:** 1 BRAC Research and Evaluation Division, 75 Mohakhali, BRAC Centre, Dhaka 1212, Bangladesh; dryunus155@gmail.com (F.M.Y.); safayet.k@brac.net (S.K.); priya.ch04@gmail.com (P.C.); 2 James P. Grant School of Public Health, BRAC University, 68 Shahid Tajuddin Ahmed Sharani, Mohakhali, Dhaka 1212, Bangladesh; 3 Centre for Clinical Epidemiology and Biostatistics (CCEB), School of Medicine and Public Health, The University of Newcastle, NSW 2308, Australia; milton.hasnat@newcastle.edu.au (A.H.M.); sumaira.87@gmail.com (S.H.)

**Keywords:** review, arsenic, Bangladesh, Millennium Development Goal

## Abstract

Arsenic contamination in drinking water has a detrimental impact on human health which profoundly impairs the quality of life. Despite recognition of the adverse health implications of arsenic toxicity, there have been few studies to date to suggest measures that could be taken to overcome arsenic contamination. After the statement in 2000 WHO Bulletin that Bangladesh has been experiencing the largest mass poisoning of population in history, we researched existing literature to assess the magnitude of groundwater arsenic contamination in Bangladesh. The literature reviewed related research that had been initiated and/or completed since the implementation of the Millennium Development Goals (MDGs) under four domains: (1) extent of arsenic contamination; (2) health consequences; (3) mitigation and technologies and (4) future directions. To this means, a review matrix was established for analysis of previous literature based on these four core domains. Our findings revealed that several high-quality research articles were produced at the beginning of the MDG period, but efforts have dwindled in recent years. Furthermore, there were only a few studies conducted that focused on developing suitable solutions for managing arsenic contamination. Although the government of Bangladesh has made its population’s access to safe drinking water a priority agenda item, there are still pockets of the population that continue to suffer from arsenic toxicity due to contaminated water supplies.

## 1. Introduction

In 2000 Bangladesh was quoted as having suffered the largest mass poisoning of a population in history according to the World Health Organisation (WHO) Bulletin. This is due to the contamination of groundwater—the main source of drinking water—with naturally occurring inorganic arsenic [1]. The number of individuals suffering from adverse health effects due to arsenic toxicity far exceeds the number of casualties from man-made disasters such as the Chernobyl nuclear disaster [2]. Arsenic poisoning via groundwater contamination has been driven by deliberate changes in human behavior. For example, combustion of coal releases arsenic from coal waste products into the environment through the process of leaching [3]. To date, there has been a paucity of conducted studies that focus on developing suitable solutions for managing arsenic contamination, thereby, the problem of access to safe drinking water persists. Recently announced Sustainable Development Goals (SDG) also highlight the importance of universal and equitable access to safe and affordable drinking water by establishing it as one of the 17 global goals (SDG 6) to be achieved by 2030 [4]. In order to determine the magnitude of groundwater arsenic contamination in Bangladesh, a thorough review of existing literature was deemed necessary. Fifteen years of literature was reviewed, encompassing research that had been initiated and/or completed since the implementation of the Millennium Development Goals (MDGs) under four domains: (1) extent of arsenic contamination; (2) health consequences; (3) mitigation and technologies and (4) future directions. As a result, a review matrix based on these four core domains was developed for analysis of the existing literature (Table 1).

## 2. A History of Arsenic

Our awareness of the presence of natural arsenic can be dated as far back as 3000 BC and humans have been using it ever since, for both malicious and altruistic purposes ranging from the use of arsenic compounds for the extraction of iron from iron ores to medicinal purposes [5]. Prior to the advent of modern antibiotics, intermediate doses (0.05 to 0.5 mg/kg/day) of arsenic were prescribed to treat patients with syphilis [6]. The use of arsenic in medicine was initiated in the 15th century by William Withering, who discovered the beneficial effects of digitalis, an arsenic-based drug [7]. Later in 18th century Thomas Fowler made a mixed compound commonly known as “Fowler’s solution” which is a potassium bicarbonate-based solution of arsenic trioxide (As_2_O_3_) that was used to treat a variety of diseases until the 20th century. There also exists evidence to suggest that arsenic was used to kill political opponents in Italy [8]. It was not until the mid-19th century that people realized that arsenic is dangerous, even at low repeated doses [6].

High concentrations of arsenic-rich ores discharged into ground and surface waters can cause damaging health effects to humans if consumed in large amounts over a long period [9]. According to WHO guidelines, water containing over 10 μg/L of arsenic is already deemed to be contaminated, but in Bangladesh this benchmark rises to 50 μg/L [10]. The Indo-Gangetic plain contains groundwater so heavily contaminated with arsenic that concentrations are over 50 μg/L in these regions of India and Bangladesh [11].

Traditionally, people in Bangladesh had used surface water sources for all their daily water needs, including drinking water. This had resulted in adverse health outcomes, for example, there was an outbreak of cholera in Bangladesh in the mid-1970s due to surface water use [6]. Microorganism contamination of surface water sources contributes significantly to the burden of infectious diseases and mortality [1]. To prevent cholera and other water-borne diseases, access to safe groundwater through tube wells was proposed as a simple and affordable solution. By the year 2000, 10 million tube wells had been sunk throughout Bangladesh [6]. However, this resulted in an unforeseen and destructive population-wide health impact—arsenic contamination of tube wells was confirmed in 1993 in the Nawabganj district of Bangladesh [11]. In 1983, Krishna Chandra Saha had identified early stage cases of arsenic-induced skin lesions in India and by 1987 he had detected several cases of arsenic-induced skin lesions in Bangladesh. However, it was only in 1993 that the Public Health Engineering Department of Bangladesh officially confirmed arsenic contamination in Nawabganj district [12,13,14]. Arsenic contamination of underground water continued to be reported from several other areas of the country, with the Government of Bangladesh (GoB) taking note of the immensity of the problem and its threat to public health in 1996 [15]. With the increasing number of tube wells over the past 40 years and their widespread use amongst the population, the problem of arsenic-contaminated groundwater continues to be of national and international concern [1].

**Table 1:** Review matrix of arsenic research during the MDG era.

Author(s)	Year	Extend of As Contamination	Health Consequences	Mitigation & Technologies	Future Directions
Chowdhury, U.K.; Biswas, B.K.; Chowdhury, T.R.; Samanta, G.; Mandal, B.K.; Basu, G.C.; Chanda, C.R.; Lodh, D.; Saha, K.C.; Mukherjee, S.K. [16].	2000		x		
Mazumder, D.N.G.; Haque, R.; Ghosh, N.; De Binay, K.; Santra, A.; Chakraborti, D.; Smith, A.H. [17].	2000		x		
Ahmad, K. [18].	2001	x		x	
World Health Organization (WHO) [19].	2001		x		
Ahmad, S.A.; Sayed, M.; Barua, S.; Khan, M.H.; Faruquee, M.; Jalil, A.; Hadi, S.A.; Talukder, H.K. [20].	2001		x		
Arcury, T.A.; Quandt, S.A.; Russell, G.B. [21].	2002			x	
Mitra, A.K.; Bose, B.K.; Kabir, H.; Das, B.K.; Hussain, M. [22].	2002	x			
Navas-Acien, A.; Sharrett, A.R.; Silbergeld, E.K.; Schwartz, B.S.; Nachman, K.E.; Burke, T.A.; Guallar, E. [23].	2005		x		
Milton, A.H.; Smith, W.; Rahman, B.; Hasan, Z.; Kulsum, U.; Dear, K.; Rakibuddin, M.; Ali, A. [24].	2005		x		
Ahamed, S.; Sengupta, M.K.; Mukherjee, S.C.; Pati, S.; Mukherjee, A.; Rahman, M.M.; Hossain, M.A.; Das, B.; Nayak, B.; Pal, A. [25].	2006	x	x	x	
Howard, G.; Ahmed, M.F.; Shamsuddin, A.J.; Mahmud, S.G.; Deere, D. [26].	2006			x	
Aziz, S.N.; Boyle, K.J.; Rahman, M. [27].	2006			x	
Caldwell, B.K.; Smith, W.T.; Lokuge, K.; Ranmuthugala, G.; Dear, K.; Milton, A.H.; Sim, M.R.; Ng, J.C.; Mitra, S. [28].	2006			x	
McDonald, C.; Hoque, R.; Huda, N.; Cherry, N. [29].	2006	**x**	**x**		
Ferreccio, C.; Sancha, A.M. [30].	2006		x		
Sun, G.; Li, X.; Pi, J.; Sun, Y.; Li, B.; Jin, Y.; Xu, Y. [31].	2006		x		
Mukherjee, A.; Sengupta, M.K.; Hossain, M.A.; Ahamed, S.; Das, B.; Nayak, B.; Lodh, D.; Rahman, M.M.; Chakraborti, D. [32].	2006	x			
Chen, Y.; Hakim, M.E.; Parvez, F.; Islam, T.; Rahman, A.M.; Ahsan, H. [33].	2006		x		
Anstiss, R.G.; Ahmed, M. [34].	2006				
Van Geen, A.; Trevisani, M.; Immel, J.; Jakariya, M.; Osman, N.; Cheng, Z.; Gelman, A.; Ahmed, K. [35].	2006			x	
Joya, S.A.; Mostofa, G.; Yousuf, J.; Islam, A.; Elahi, A.; Mahiuddin, G.; Rahman, M.; Quamruzzaman, Q.; Wilson, R. [36].	2006			x	
Wasserman, G.A.; Liu, X.; Parvez, F.; Ahsan, H.; Factor-Litvak, P.; Kline, J.; Van Geen, A.; Slavkovich, V.; Lolacono, N.J.; Levy, D. [37].	2007		x		
Rahman, A.; Vahter, M.; Ekström, E.-C.; Rahman, M.; Mustafa, A.H.M.G.; Wahed, M.A.; Yunus, M.; Persson, L.-Å. [38].	2007		x		
Ravenscroft, P.; Brammer, H.; Richards, K. [39].	2009	x			
Khan, N.I.; Bruce, D.; Naidu, R.; Owens, G. [40].	2009		x		
Argos, M.; Kalra, T.; Rathouz, P. J.; Chen, Y.; Pierce, B.; Parvez, F.; Islam, T.; Ahmed, A.; Rakibuz-Zaman, M.; Hasan, R.; Sarwar, G.; Slavkovich, V.; van Geen, A.; Graziano, J.; Ahsan, H. [41].	2010		x		
Chen, Y.; Graziano, J.H.; Parvez, F.; Liu, M.; Slavkovich, V.; Kalra, T.; Argos, M.; Islam, T.; Ahmed, A.; Rakibuz-Zaman, M. [42].	2011		x		
Argos, M.; Kalra, T.; Pierce, B. L.; Chen, Y.; Parvez, F.; Islam, T.; Ahmed, A.; Hasan, R.; Hasan, K.; Sarwar, G.; Levy, D.; Slavkovich, V.; Graziano, J. H.; Rathouz, P. J.; Ahsan, H. [43].	2011		x		
Ahmed, S.; Mahabbat-e Khoda, S.; Rekha, R. S.; Gardner, R. M.; Ameer, S. S.; Moore, S.; Ekström, E.-C.; Vahter, M.; Raqib, R. [44].	2011		x		
Rahman, A.; Vahter, M.; Ekström, E.-C.; Persson, L.-Å. [45].	2011		x		
Flanagan, S.V.; Johnston, R.B.; Zheng, Y. [46].	2012			x	
George, C.M.; van Geen, A.; Slavkovich, V.; Singha, A.; Levy, D.; Islam, T.; Ahmed, K.M.; Moon-Howard, J.; Tarozzi, A.; Liu, X. [47].	2012			x	
Milton, A.H.; Hore, S.K.; Hossain, M.Z.; Rahman, M. [15].	2012		x	x	x
Argos, M.; Ahsan, H.; Graziano, J. H., Arsenic and human health: epidemiologic progress and public health implications. [48].	2012				x
Adams, P. [49].	2013		x		
Chen, Y.; Wu, F.; Graziano, J. H.; Parvez, F.; Liu, M.; Paul, R. R.; Shaheen, I.; Sarwar, G.; Ahmed, A.; Islam, T.; Slavkovich, V.; Rundek, T.; Demmer, R. T.; Desvarieux, M.; Ahsan, H. [50].	2013		x		
Argos, M.; Melkonian, S.; Parvez, F.; Rakibuz-Zaman, M.; Ahmed, A.; Chen, Y.; Ahsan, H. [51].	2013		x		
Yunus, F.M.; Rahman, M.J.; Alam, M.Z.; Hore, S.K.; Rahman, M. [52].	2014		x		
Khan, N.I.; Yang, H. [53].	2014			x	
Argos, M.; Parvez, F.; Rahman, M.; Rakibuz-Zaman, M.; Ahmed, A.; Hore, S. K.; Islam, T.; Chen, Y.; Pierce, B. L.; Slavkovich, V.; Olopade, C.; Yunus, M.; Baron, J. A.; Graziano, J. H.; Ahsan, H. [54].	2014		x		
Rahman, M.; Sohel, N.; Hore, S. K.; Yunus, M.; Bhuiya, A.; Streatfield, P. K. [55].	2015	x			
Wu, Y.; Li, W.; Sparks, D. L. [56].	2015			x	
Pesola, G. R.; Argos, M.; Chen, Y.; Parvez, F.; Ahmed, A.; Hasan, R.; Rakibuz-Zaman, M.; Islam, T.; Eunus, M.; Sarwar, G.; Chinchilli, V. M.; Neugut, A. I.; Ahsan, H. [57].	2015		x		

## 3. Extent of Arsenic Contamination in Bangladesh

Almost 57 million people in Bangladesh are at risk of arsenic-induced disease due to chronic contamination of their drinking water with arsenic concentrations exceeding the WHO limit [18]. Contamination of shallow tube wells has resulted in 80% of the Bangladeshi population being exposed to arsenic as the groundwater was used for their daily cooking and drinking needs [24]. Large-scale arsenic poisoning amongst rural people is further compounded by chronic undernutrition and poverty. Arsenic poisoning has significant economic implications as it affects agriculture, water management and public health [18]. A study on arsenic concentrations in tube-well water in Barisal (Bangladesh) examined 150 patients, revealing that of the 25% of patients that used drinking water from contaminated tube wells, 82% had moderate or severe skin lesions characteristic of arsenic exposure. Moreover, 31% of water samples had 10-fold higher arsenic concentrations greater than the acceptable limit of 50 μg/L in Bangladesh and a 50-fold higher concentration than the WHO recommended value of 10 μg/L [22].

The presence of arsenic contamination throughout Bangladesh is extensive, as evidenced through the findings in 1998, where 41 of the 64 districts in Bangladesh had concentrations of arsenic in groundwater exceeding 50 μg/L [32]. By 2005, another study reported that 50 of 64 districts in Bangladesh had groundwater arsenic concentrations exceeding 50 μg/L [25]. The groundwater arsenic found in the aquifer sediments in Bangladesh is found to be controlled by Fe- and Mn-oxyhydroxides under anoxic conditions [58]. The concentration of arsenic varies within different depth of the ground and is dependent upon the surface geology and hydrogeological conditions of the area. The concentration is found to be higher at depths of 20–70 m from the surface [59]. Numerous literature reports have highlighted that a higher arsenic concentration in groundwater is associated with a higher prevalence of arsenicosis [29]. A study in the village of Eruani, Comilla stated that 25% of the sample population had arsenic-associated skin lesions [25]. The GoB now considers this a national public health problem because an estimated 27 million and 50 million people have been exposed to arsenic through drinking water containing over 50 µg/L and 10 µg/L of arsenic in Bangladesh [39].

Although it is a well-known fact that drinking arsenic contaminated ground water is a potential source for arsenic poisoning in humans, there are other sources, including ingestion of arsenic- contaminated food. A study examined three successive routes (water-soil-crop) of arsenic contamination in the food chain and found increased levels of arsenic in crops commonly grown in Bangladesh, including different varieties of rice. Furthermore, this study argues that even if pure drinking water is secured, arsenic-contaminated ground water would still be used for irrigation purposes which still poses a significant threat to human health [60]. Bangladeshi population thus also gets exposed to arsenic through the ingestion of rice, which is the staple food. Paddy soils in Bangladesh are found to contain high levels of arsenic because of irrigation with arsenic-contaminated water [61]. The concentration of arsenic was also found to be higher in cooked rice when compared to raw rice with absorbed water [62]. In both cases, it may be because rice is naturally more susceptible to accumulate higher concentrations of arsenic compared to other cereals [63]. In Bangladesh, most of the people eat rice that is cooked twice: first, the paddy is boiled with possibly arsenic contaminated water, which is then dried off to make rice. Second the dried rice is cooked again with large quantities of water. The two step rice preparation process may increase the quantity of arsenic in the final product [64].

## 4. Health Consequences of Arsenic Exposure (Table 2)

Arsenic exposure has severe health consequences and has been recognized as a major public health concern across the world over the last two to three decades. Arsenic-associated skin problems were first recognized in Bangladesh in 1995, but it was not until 1998 that public attention was drawn to these problems [16]. Often the early symptoms of chronic arsenic exposure include skin darkening (diffuse or spotted melanosis) over the chest, back or other parts of the body. Drinking water concentration of less than 10 μg/L has been proven to be associated with increased risk of skin lesion incidence and these lesions exist even after the patient has reduced the arsenic exposure in recent years [43] Arsenic exposure results in characteristic dermal effects starting with melanosis (pigmentation) followed by keratosis and hyperkeratosis [25,65]. These dermal features herald arsenic toxicity, which occurs when melanosis and keratosis appear together [25]. In addition to these, there is an increased risk of lung cancer mortality among persons with arsenical skin lesions and also by the severity of their skin lesions [54] Arsenic exposure can cause hand and foot lesions, extending to cause disability in some cases. Besides characteristic dermal lesions (spotted pigmentation, leucomelanosis, mucus membrane melanosis, spotted and diffuse keratosis, palpable nodules on the dorsum of the hands, feet, and legs) the symptoms of arsenic toxicity include conjunctival congestion, non-pitting foot swelling, hepatomegaly, splenomegaly, ascites, Bowen’s disease and gangrene [25]. Apart from these, increasing concentration of arsenic in well water was found to be associated with increased Carotid-intima Media Thickness (CMT) [50]. The dietary preference of the population, particularly the heavy intake of ground or root vegetables by the Bangladeshi population, is also found to be positively associated with CMT [66].

A large-scale longitudinal study conducted in Bangladesh reported that high-concentration arsenic exposure could have both carcinogenic and non-carcinogenic effects [17,19,37,38]. Non-carcinogenic effects include chronic diseases such as vascular diseases, liver disease, neurotoxicity, chronic cough and diabetes mellitus. High concentrations of arsenic exposure can cause adverse pregnancy outcomes and impair child development. In terms of carcinogenic effects, exposed to high arsenic exposure concentrations increase the risk of developing cancer, a risk that persists for decades after initial arsenic exposure and its withdrawal [67]. Consumption of and exposure to arsenic can also cause various types of cancer, including lung and bladder cancer [15,30,31]. Although Bangladesh-specific data on lung cancer, bladder cancer, and teratogenicity are not yet available [29], a recent population based cohort study conducted in Araihazar (Bangladesh) has shown a positive association between increased uptake of dietary energy, especially protein, and mortality from cancers. The highest risk of mortality in adults was in cancers of the digestive system [51].

A number of studies concerning the effects of arsenic on maternal health and their unborn child were conducted on pregnant women. There is a positive association between arsenic exposure via drinking water and spontaneous abortions [20,24,25], stillbirths [20,24,25], and pre-term births [20,25]. Enhanced placental inflammatory responses, reduced placental T-cells counts, and changed cord blood cytokines appear to all be caused by maternal arsenic exposure during pregnancy [44]. Observations from arsenic-affected areas of Bangladesh confirmed different findings related to child health, particularly with respect to childrens’ low birth weight [20,25], neonatal mortality [24,25] and infant mortality [24,25]. Prenatal arsenic exposure is also found to be associated with increased risk of morbidity in infectious diseases such as Lower Respiratory Tract Infection (LRTI) and diarrhea during infancy (<5 years age) [45]. Furthermore, arsenic exposure during pregnancy hampers the neurodevelopment of children, which continues years after birth and has also been associated with increased risk of 1-5 year aged children drowning [55]. With respect to female reproduction, it has been shown that arsenic skin lesions are associated with early-onset menopause, with chronic high arsenic exposure resulting in a two-year earlier menopause and reduced reproductive period [52].

**Table 2:** Review matrix of health consequences of arsenic exposure.

Author(s)	Study Title	Year	Carcinogenic	Non-Carcinogenic
Mazumder, D.N.G.; Haque, R.; Ghosh, N.; De Binay, K.; Santra, A.; Chakraborti, D.; Smith, A.H. [17]	Arsenic in drinking water and the prevalence of respiratory effects in west Bengal, India.	2000	x	x
Ahmad, K. [18]	Report highlights widespread arsenic contamination in Bangladesh.	2001	x	x
Ahmad, S.A.; Sayed, M.; Barua, S.; Khan, M.H.; Faruquee, M.; Jalil, A.; Hadi, S.A.; Talukder, H.K. [20].	Arsenic in drinking water and pregnancy outcomes.	2001		x
World Health Organisation (WHO) [19].	Environmental Health Criteria 224: Arsenic and Arsenic Compounds	2001	x	x
Mitra, A.K.; Bose, B.K.; Kabir, H.; Das, B.K.; Hussain, M. [22].	Arsenic-related health problems among hospital patients in southern Bangladesh.	2002	x	x
Milton, A.H.; Smith, W.; Rahman, B.; Hasan, Z.; Kulsum, U.; Dear, K.; Rakibuddin, M.; Ali, A. [24].	Chronic arsenic exposure and adverse pregnancy outcomes in Bangladesh.	2005		x
Navas-Acien, A.; Sharrett, A.R.; Silbergeld, E.K.; Schwartz, B.S.; Nachman, K.E.; Burke, T.A.; Guallar, E. [23].	Arsenic exposure and cardiovascular disease: A systematic review of the epidemiologic evidence.	2005		x
Ahamed, S.; Sengupta, M.K.; Mukherjee, S.C.; Pati, S.; Mukherjee, A.; Rahman, M.M.; Hossain, M.A.; Das, B.; Nayak, B.; Pal, A. [25].	An eight-year study report on arsenic contamination in groundwater and health effects in eruani village, Bangladesh and an approach for its mitigation.	2006		x
McDonald, C.; Hoque, R.; Huda, N.; Cherry, N. [29].	Prevalence of arsenic-related skin lesions in 53 widely-scattered villages of Bangladesh: An ecological survey.	2006		x
Ahsan, H.; Chen, Y.; Parvez, F.; Zablotska, L.; Argos, M.; Hussain, I.; Momotaj, H.; Levy, D.; Cheng, Z.; Slavkovich, V. [68].	Arsenic exposure from drinking water and risk of premalignant skin lesions in Bangladesh: Baseline results from the health effects of arsenic longitudinal study.	2006	x	
Ferreccio, C.; Sancha, A.M. [30].	Arsenic exposure and its impact on health in chile.	2006	x	
Sun, G.; Li, X.; Pi, J.; Sun, Y.; Li, B.; Jin, Y.; Xu, Y. [31].	Current research problems of chronic arsenicosis in china.	2006	x	
Chen, Y.; Hakim, M.E.; Parvez, F.; Islam, T.; Rahman, A.M.; Ahsan, H. [33].	Arsenic exposure from drinking-water and carotid artery intima-medial thickness in healthy young adults in Bangladesh.	2006		x
Rahman, A.; Vahter, M.; Ekström, E.-C.; Rahman, M.; Mustafa, A.H.M.G.; Wahed, M.A.; Yunus, M.; Persson, L.-Å. [38].	Association of arsenic exposure during pregnancy with fetal loss and infant death: A cohort study in Bangladesh.	2007	x	x
Wasserman, G.A.; Liu, X.; Parvez, F.; Ahsan, H.; Factor-Litvak, P.; Kline, J.; Van Geen, A.; Slavkovich, V.; Lolacono, N.J.; Levy, D. [37].	Water arsenic exposure and intellectual function in 6-year-old children in araihazar, Bangladesh.	2007	x	x
Khan, N.I.; Bruce, D.; Naidu, R.; Owens, G. [40].	Implementation of food frequency questionnaire for the assessment of total dietary arsenic intake in Bangladesh: Part b, preliminary findings.	2009		x
Argos, M.; Kalra, T.; Rathouz, P. J.; Chen, Y.; Pierce, B.; Parvez, F.; Islam, T.; Ahmed, A.; Rakibuz-Zaman, M.; Hasan, R.; Sarwar, G.; Slavkovich, V.; van Geen, A.; Graziano, J.; Ahsan, H. [41].	Arsenic exposure from drinking water, and all-cause and chronic-disease mortalities in Bangladesh (HEALS): a prospective cohort study.	2010		x
Chen, Y.; Graziano, J.H.; Parvez, F.; Liu, M.; Slavkovich, V.; Kalra, T.; Argos, M.; Islam, T.; Ahmed, A.; Rakibuz-Zaman, M. [42].	Arsenic exposure from drinking water and mortality from cardiovascular disease in Bangladesh: Prospective cohort study.	2011		x
Argos, M.; Kalra, T.; Pierce, B. L.; Chen, Y.; Parvez, F.; Islam, T.; Ahmed, A.; Hasan, R.; Hasan, K.; Sarwar, G.; Levy, D.; Slavkovich, V.; Graziano, J. H.; Rathouz, P. J.; Ahsan, H. [43].	A prospective study of arsenic exposure from drinking water and incidence of skin lesions in Bangladesh.	2011		x
Ahmed, S.; Mahabbat-e Khoda, S.; Rekha, R. S.; Gardner, R. M.; Ameer, S. S.; Moore, S.; Ekström, E.-C.; Vahter, M.; Raqib, R., [44].	Arsenic-associated oxidative stress, inflammation, and immune disruption in human placenta and cord blood.	2011		x
Rahman, A.; Vahter, M.; Ekström, E.-C.; Persson, L.-Å., [45].	Arsenic exposure in pregnancy increases the risk of lower respiratory tract infection and diarrhea during infancy in Bangladesh	2011		x
Milton, A.H.; Hore, S.K.; Hossain, M.Z.; Rahman, M. [15].	Bangladesh arsenic mitigation programs: Lessons from the past.	2012		x
Adams, P. [49].	In Bangladesh, funds dry up for arsenic mitigation research.	2013		x
Chen, Y.; Wu, F.; Graziano, J. H.; Parvez, F.; Liu, M.; Paul, R. R.; Shaheen, I.; Sarwar, G.; Ahmed, A.; Islam, T.; Slavkovich, V.; Rundek, T.; Demmer, R. T.; Desvarieux, M.; Ahsan, H. [50].	Arsenic exposure from drinking water, arsenic methylation capacity, and carotid intima-media thickness in Bangladesh	2013		x
Argos, M.; Melkonian, S.; Parvez, F.; Rakibuz-Zaman, M.; Ahmed, A.; Chen, Y.; Ahsan, H. [51].	A population-based prospective study of energy-providing nutrients in relation to all-cause cancer mortality and cancers of digestive organs mortality.	2013	x	
Yunus, F.M.; Rahman, M.J.; Alam, M.Z.; Hore, S.K.; Rahman, M. [52].	Relationship between arsenic skin lesions and the age of natural menopause.	2014		x
Argos, M.; Parvez, F.; Rahman, M.; Rakibuz-Zaman, M.; Ahmed, A.; Hore, S. K.; Islam, T.; Chen, Y.; Pierce, B. L.; Slavkovich, V.; Olopade, C.; Yunus, M.; Baron, J. A.; Graziano, J. H.; Ahsan, H. [54].	Arsenic and lung disease mortality in Bangladeshi adults	2014	x	x
Rahman, M.; Sohel, N.; Hore, S. K.; Yunus, M.; Bhuiya, A.; Streatfield, P. K. [55].	Prenatal arsenic exposure and drowning among children in Bangladesh	2015		x
Pesola, G. R.; Argos, M.; Chen, Y.; Parvez, F.; Ahmed, A.; Hasan, R.; Rakibuz-Zaman, M.; Islam, T.; Eunus, M.; Sarwar, G.; Chinchilli, V. M.; Neugut, A. I.; Ahsan, H, [57].	Dipstick proteinuria as a predictor of all-cause and cardiovascular disease mortality in Bangladesh: A prospective cohort study.	2015		x

A positive association was also found between long term arsenic exposure and blood pressure increase over-time which might lead to cardiovascular diseases [68]. Genetic variability also contributes to arsenic associated increases in blood pressure (BP) over time [69]. High arsenic exposure from drinking water (50–690 μg/L and >690 μg/L compared to <50 μg/L) has also been associated with early carotid atherosclerosis in an older population in Taiwan. The study assessed the relative risk of highest arsenic exposure to lowest for coronary diseases (1.59 to 4.90), stroke (1.19 to 2.69) and peripheral arterial disease (1.66 to 4.28) [23]. Besides these studies, Chen *et al.* found an association between the amount of arsenic exposure and carotid artery wall thickening [33]. After an average of 6.6 years, the cardiovascular mortality rate was 214.3 per 100,000 person years in people drinking water containing <12.0 µg/L arsenic compared to 271.1 per 100,000 person years in people drinking water with ≥12.0 µg/L arsenic [42]. The Health Effects of Arsenic Longitudinal Cohort Study (HEALS) in Bangladesh also reported the association of chronic arsenic exposure in drinking water with an increase in the all-cause mortality and chronic disease mortality rate [41]. This all-cause or chronic disease mortality can be caused by dipstick proteinuria which was found to be associated with increasing arsenic exposure [70]. Different cardiovascular risk factors such as diabetes mellitus, hypertension, increased BMI are associated with dipstick proteinuria which is again responsible for all-cause mortality or cardio-vascular disease mortality in developing countries including Bangladesh [57]. In another study, Chen *et al.* showed that there was a greater risk of mortality from heart disease due to the joint effect of moderate arsenic exposure and cigarette smoking than their individual effects alone and there was greater carotid wall thickening in men than in women [33]. Furthermore, it has been recently shown that eating food crops irrigated by contaminated groundwater, including rice, might place individuals at increased risk of death and disease [40,49]. As illustrated, the list of adverse health consequences due to arsenic exposure is lengthy, ranging from pregnancy complications, cardiovascular morbidity to carcinogenic effects. Arsenic toxicity not only increases morbidity but also creates social issues that can lead to severe discrimination. Typically, arsenic-induced hyper-pigmentation and hyperkeratosis is widely distributed over the trunk and extremities, which inhibits patients from participating in social activities. Alarmingly, in one study, a quarter of all arsenic patients stopped participating in social activities and women were the most severely affected [71].

## 5. Mitigation and Technologies

Numerous alternative water supply technologies have been identified and tested in different areas of Bangladesh to reduce the concentration of arsenic in water. The GoB started implementing mitigation programs in 1996 with support from developmental partners as well as national and international NGOs [15]. Improved wells, deep tube wells, pond-sand filters, rainwater harvesting and piped water supplies have all been piloted and adopted in Bangladesh as possible mitigation strategies [15,25,26]. Knowledge building by means of campaigns and surveys was initially used to raise awareness about arsenic contamination and its effects. Some people were unaware of the link between contaminated water and diarrhea in Bangladesh. It is important to increase awareness about the link between water and disease risk in developing countries so that the population can demand safe drinking water [27]. It has been shown that perceived risk may decrease with increased knowledge [21], therefore, it is important for the target population to understand the extent of the problem and agree to adopt alternative options to mitigate risk and demand safe water supplies [27]. Fortunately, awareness of arsenic in Bangladesh has increased since 2000, particularly in women, reflecting efforts made by NGOs to increase communication and discussions between friends and neighbors [28]. With the success of awareness campaigns, people appear to be more willing to walk long distances to avoid exposure to arsenic if the arsenic-free water source is a tube well rather than surface water, since surface water requires further avoidance measures such as boiling. However, as illustrated here, convenience is an important factor to consider in public health mitigationpolicies and measures to ensure that people avoid the health risks associated with consuming pathogen-contaminated surface water [27].

Mitigation strategies also involve testing tube wells and providing alternative water supply options such as fetching water from dug wells or trapping rain water. It has been found that very shallow wells (less than 10 m deep) and the deeper wells (more than 150 m) are not contaminated with arsenic [59], so, precautions should be taken during installation of any new tube-well within this depth range. The GoB Department of Public Health Engineering’s (DPHE) first nationwide public and private tube-well screening project was supported by UNICEF, with screening taking place in arsenic prone areas and prompting mainstream arsenic mitigation in general water and sanitation programs [72]. Other studies have shown that, after performing the thermo-tolerant coliform test for fecal contamination in water using field testing kits, dug wells and pond-sand filters were effective at eliminating microbes during both the dry and rainy seasons (minimum count of <1 cfu/100 mL). Rainwater harvesting was shown to produce the best microbial quality, however, regular contamination existed, particularly in the dry season where Bangladeshi standards were exceeded by 60% [26]. As an alternative, deep tube wells can be used in the dry season as they are of good quality overall and are more robust to microbial contamination [26,34,53], despite being more contaminated during the monsoon season [60]. If there is insufficient rain in the dry season and no arsenic-free deep aquifer has been found or is not favored, the most technically-feasible option that remains is to increase the number of arsenic treatment devices on tube wells. It is then important to re-assess the suitability of the drinking-water configuration depending upon the location and region [34].

In the fast evolving world of technology, new techniques can be applied to provide access to safe drinking water. For instance, mobile phone technology was used in Araihazar to locate the optimal start depth for installing safe tube wells. In this system, the mobile phone is connected to a laptop on which SMS requests are handled by a robust open source software model database server called jSMSEngine/MySQL. The software runs a search algorithm programmed in R that queries the database and estimates the start depth, with the response sent back to the mobile phone via SMS [35]. Arsenic mitigation for the provision of safe water needs to be prioritized for an estimated 5 million people exposed to over 200 μg/L arsenic [15,46]. Some mitigation strategies rely on building local arsenic testing capacity but this utility needs to be further capitalized as it leads to sustained awareness in the most affected areas. Furthermore, it provides the incentive to regularly test wells and undertake arsenic mitigation measures. For example, installing safe water points with no more than 50 people per point and providing technologies that avoid rather than remove arsenic contamination, provide long term cost effectiveness [46]. This pattern is repeated in several studies, demonstrating that arsenic education and water testing is an effective, low-cost approach for reducing arsenic exposure in many arsenic affected areas in Bangladesh [27,47]. Residents in areas where the ground water is contaminated with arsenic used various conventional technologies to reduce arsenic concentration in drinking water. These technologies include removal of arsenic using co-precipitation with ferric chloride, lime softening and filtration using exchange resin. Research also found that adding Mn-oxides and Fe(II) to arsenic contaminated water helps to remove arsenic from drinking water [56]. Often people use adsorbents such as alumina and membrane filtration [73,74,75]. This is utilized by adding a packet of 2 g ferric and hydrochloride salt to 20 L water and using a bucket sand filter to repeatedly filter the water [76]. Studies showed that conventional techniques such as co-precipitation and filtration are simple and affordable methods to reduce arsenic contamination in water, costing less that U.S. $4 annually per family to drink 50 L of filtered arsenic-free water daily [36].

Further research needs to be undertaken to find the minimum safe threshold of arsenic so that sustainable mitigation strategies can be tailored. However, research funding for sustainable mitigation options is difficult to obtain [49]. Also there is lack of support from governmental organizations and local stakeholders as well as absence of a local market where arsenic mitigation technologies are readily accessible to local communities [53]. A greater cohesive commitment is required from the government and international development agencies to address these issues.

## 6. Future Directions

It is evident that arsenic contamination in water has an expansive impact on health and can impair quality of life. Although a number of high-quality research articles were published after the development of the MDGs, unfortunately, less research has been undertaken in recent years. It might be beneficial to adopt a holistic approach to minimizing the causes and effects of arsenic contamination. As the MDG expires in 2015, the UN General Assembly’s Open Working Group on Sustainable Development Goals (SDGs) drafted a set of goals in July 2014 to improve quality of life. Of the 17 targeted goals, SDG 2 (end hunger, achieve food security and improve nutrition, and promote sustainable agriculture), SDG 3 (ensure healthy lives and promote well-being for all at all ages) and SDG 6 (ensure access to water and sanitation for all) are closely interlinked with the problem of arsenic contamination.

### 6.1. Research

Despite there being a number of methods to minimize arsenic contamination, little is known about the most cost-effective way of dealing with groundwater arsenic contamination. Questions that still remain include: How many people use arsenic concentration testing devices? Can they operate them? If they use them, how long do they use them for? Which family member makes the decision to use such a device? Are people aware that reducing visible arsenic lesions takes a long time? How effective are medicines or supplements in reducing arsenic toxicity? What are their priority health consequences? What is the government policy for mitigating arsenic contamination? Are other partners pushing this issue at policy level? Are there sufficient funds to tackle this issue? Answers to these questions will offer greater insight into how to deal with the issue of arsenic contaminated water in Bangladesh.

Behavioral research needs to be conducted to determine effective methods to mitigate arsenic exposure. This is especially important considering the fact that chronic exposure to arsenic increases risk of morbidity and mortality, with the risk not decreasing even after measures have been taken to reduce arsenic exposure [41]. For example, due to lack of convenience, people residing in areas of high arsenic exposure prefer not to travel long distances from their home to collect drinking water from tube wells. Especially when some tube wells, marked as green 20 years ago to signify safe for drinking, have now been contaminated with arsenic. Another research avenue worth investigating includes genetic and molecular pathways of arsenic related health outcomes. This may provide future secondary prevention strategies for arsenic exposed populations [48].

### 6.2. Policy Level

Arsenic contamination in drinking water requires more technical attention, since it has long been declared a national public health problem in Bangladesh. The problem needs to be understood at the policy level, with public health experts taking on a leading role in arsenic mitigation programs to ensure that they are effective. Health prevention and promotion strategies in conjunction with remediation of arsenic exposed populations should go hand-in-hand [48]. Region-specific water supply options are needed, since there are variations in geo-hydrology, soil types and water chemistry. More data is required on these factors and their effect on implementing arsenic removal technologies and their outcomes [15].

### 6.3. Public Health Programs

Every public health intervention, ranging from Maternal, Neonatal and Child Health (MNCH) to human rights, needs to include arsenic awareness messages and ways to mitigate arsenic contamination. The detrimental health consequences of arsenic contamination need to be more widely recognized. In general, people are only aware of the short-term visible health effects, particularly the poverty stricken population who struggle to survive each day. Arsenic mitigation programs should be developed in such a manner that the community can actively engage with the process from planning to implementation, so that ultimately they can resolve the issue themselves. This may only be possible when programs act as a bridge between the community and local government.

## 7. Conclusions

Arsenic toxicity affects millions of people in Bangladesh but surprisingly, still remains a neglected public health concern. Although the government of Bangladesh has made it a priority agenda for the population to have access to safe drinking water, there are still population pockets that continue to suffer from arsenic toxicity due to contaminated water supplies. These areas need to be identified on an urgent basis to provide comprehensive mitigation support. A governmental and non-governmental collaboration along with public-private collaboration could facilitate the mitigation efforts more effectively. Although high-quality research in this area was conducted in the early nineties, there has been a dramatic decline in useful research in this area in the latter part of the MDG period. Furthermore, there were only a few studies conducted that focused on developing suitable solutions for managing arsenic contamination. Immediate public health action from stakeholders including the government, NGOs and donors is required to reduce the burden of chronic disease caused by arsenic exposure in Bangladesh.
